# The ArcAB two-component regulatory system promotes resistance to reactive oxygen species and systemic infection by *Salmonella* Typhimurium

**DOI:** 10.1371/journal.pone.0203497

**Published:** 2018-09-04

**Authors:** Coral Pardo-Esté, Alejandro A. Hidalgo, Camila Aguirre, Alan C. Briones, Carolina E. Cabezas, Juan Castro-Severyn, Juan A. Fuentes, Cecilia M. Opazo, Claudia A. Riedel, Carolina Otero, Rodrigo Pacheco, Miguel A. Valvano, Claudia P. Saavedra

**Affiliations:** 1 Laboratorio de Microbiología Molecular, Departamento de Ciencias Biológicas, Facultad de Ciencias de la Vida, Universidad Andres Bello, Santiago, Chile; 2 Laboratorio de Patogenesis Bacteriana, Facultad de Medicina, Universidad Andres Bello, Santiago, Chile; 3 Laboratorio de Genética y Patogénesis Bacteriana, Departamento de Ciencias Biológicas, Facultad de Ciencias de la Vida, Universidad Andres Bello, Santiago, Chile; 4 Millennium Institute on Immunology and Immunotherapy, Departamento de Ciencias Biológicas, Facultad de la Vida y Facultad de Medicina, Universidad Andres Bello, Santiago, Chile; 5 Center for Integrative Medicine and Innovative Science (CIMIS), Facultad de Medicina, Universidad Andres Bello, Santiago, Chile; 6 Laboratorio de Neuroinmunología, Fundación Ciencia & Vida, Santiago, Chile; 7 Departamento de Ciencias Biológicas, Facultad de Ciencias de la Vida, Universidad Andres Bello, Santiago, Chile; 8 The Wellcome-Wolfson Institute for Experimental Medicine, Queen's University Belfast, Belfast, United Kingdom; Robert Koch Institute, GERMANY

## Abstract

*Salmonella enterica* Serovar Typhimurium (*S*. Typhimurium) is an intracellular bacterium that overcomes host immune system barriers for successful infection. The bacterium colonizes the proximal small intestine, penetrates the epithelial layer, and is engulfed by macrophages and neutrophils. Intracellularly, *S*. Typhimurium encounters highly toxic reactive oxygen species including hydrogen peroxide and hypochlorous acid. The molecular mechanisms of *Salmonella* resistance to intracellular oxidative stress is not completely understood. The ArcAB two-component system is a global regulatory system that responds to oxygen. In this work, we show that the ArcA response regulator participates in *Salmonella* adaptation to changing oxygen levels and is also involved in promoting intracellular survival in macrophages and neutrophils, enabling *S*. Typhimurium to successfully establish a systemic infection.

## Introduction

Phagocytic cells play critical roles defending the host against pathogens. However, several microorganisms, such as *Salmonella enterica* serovar Typhimurium (*S*. Typhimurium), can survive and multiply in phagocytic cells including monocytic macrophages and neutrophils [1–3].

*S*. Typhimurium survives intracellularly in a membrane-bound compartment known as the *Salmonella*-containing vacuole (SCV) [2, 4]. *Salmonella* residing inside macrophages can be protected against more lethal neutrophils and inflammatory monocytes [5]. However, despite infection of macrophages and neutrophils appear to be host vulnerable points because pathogens have the capacity to use them to their advantage, they also play a critical role in host protection [6–10]. Indeed, mice lacking neutrophils cannot control *S*. Typhimurium proliferation [11].

Reactive oxygen species (ROS) are the primary microbicides within macrophages and neutrophils [8, 12], particularly under acidic conditions. Two molecules of superoxide (O_2_^-^) react non-enzymatically to generate hydrogen peroxide (H_2_O_2_) [13] reaching concentrations between 1–4 μM to 2 mM in the phagosome [8,14]. Further, the slightly alkaline environment in neutrophils triggers the expression and activation of myeloperoxidase [14,15], which catalyzes the production of up to 73 mM hypochlorous acid (HOCl) from H_2_O_2_ and chloride [16].

*Salmonella* modulates gene expression in response to the intracellular lifestyle. Transcriptome analyses of *S*. Typhimurium collected from infecting macrophages showed that the expression of 919 of 4451 open reading frames becomes significantly altered, and 44% represented coding sequences of unknown function [6]. Similarly, 42% and 31% of *Salmonella* genes were differentially expressed when infecting macrophages or epithelial cells, respectively [17]. In particular, chlorine-based oxidative stress altered the expression of *Salmonella* genes encoding proteins implicated in the iron-sulfur cluster assembly, cysteine biosynthesis, stress response, biofilm formation and central metabolism [18].

The ROS response by *S*. Typhimurium and other enteric bacteria involves the transcription factors SoxRS and OxyR [19–22]. SoxRS, HypT, NemR, and RclR are also specifically implicated in the response to HOCl [23–26]. However, OxyR, which regulates the expression of many genes encoding proteins required for ROS detoxification, does not completely protect *S*. Typhimurium against the oxidative burst generated in phagocytic cells [27].

Several *S*. Typhimurium porins (OmpD, OmpW, OmpC, OmpS2, and OmpF) mediate H_2_O_2_ and HOCl influx. Expression of these porins is also down-regulated in response to H_2_O_2_ and HOCl, and their inactivation increases *Salmonella* ROS resistance [28, 29]. Accordingly, the absence of OmpD increases *S*. Typhimurium invasion and proliferation in RAW 264.7 macrophages, as well as proliferation and systemic dissemination in BALB/c mice [30]. We have previously ruled out a role for SoxS and OxyR in the negative regulation of porin genes; instead, the expression of several of these porins was regulated by ArcA [28, 29], a transcription factor in the ArcAB two-component system. ArcAB, which comprises the ArcA response regulator and the sensor histidine kinase ArcB, responds to oxygen [31–33]. ArcAB modulates cellular metabolism, biosynthesis and motility of anaerobically and aerobically grown *S*. Typhimurium [34, 35]. It is also required for virulence in *Haemophilus influenza*, *Vibrio cholerae*, *Actinobacillus pleuropneumoniae*, and *S*. Typhimurium [36–39].

When *S*. Typhimurium responds to H_2_O_2_ during aerobic growth, ArcA regulates gene expression and contributes to control the levels of glutathione, NADH, and intracellular ROS, as well as the pyruvate dehydrogenase complex and other processes, thus modulating the redox potential of the cell [35]. In this study, we examined in more detail the function of ArcAB two-component system in *Salmonella* pathogenesis, especially its role in *S*. Typhimurium infectivity. Our results show that ArcA participates in the bacterial adaptation to changing oxygen levels, and also promotes intracellular survival in macrophages and neutrophils, hence contributing to systemic infection.

## Materials and methods

### Ethics statement

Animals were manipulated following the recommendations in the Guide for the Care and Use of Laboratory Animals of the US National Institutes of Health, and the protocol was approved by the Bioethics Committee of Universidad Andrés Bello, Protocols 001/2012 and 06/2016 in the framework of FONDECYT Grants #1120384 and #1160315.

### Bacterial strains and growth conditions

The *S*. Typhimurium 14028s parental strain, Δ*arcA*, Δ*arcB*, Δ*arcA*/pBR::*arcA* and Δ*arcB/*pBR::*arcB*, complemented with plasmid pBR322 containing the promoter and coding regions for *arcA* and *arcB*, were maintained on LB agar plates in aerobiosis unless otherwise indicated (Table 1).

**Table 1:** Bacterial strains used in this study.

Strain	Characteristics	Source
Stm 14028s	Wild type strain of *Salmonella enterica* serovar Typhimurium 14028s ATCC	G. Mora
Δ*arcA*	Stm 14028s Δ*arcA*::*aph*	[28]
Δ*arcB*	*S*. Typhimurium Δ*arcB*::*caf*	[29]
Δ*slyA*	*S*. Typhimurium Δ*slyA*::*aph*	[46]

### Cell line cultures

RAW 264.7 (ATCC^®^ TIB-71™) murine macrophages and HEp-2 (ATCC^®^ CCL-223™) human epithelial cells were cultivated in 25-cm^2^ tissue culture flasks (Becton Dickinson Labware) with 5 ml of the specific culture media for each cell type. The Roswell Park Memorial Institute (RPMI) medium and Dulbecco’s Modified Eagle Medium (DMEM) were used for epithelial cells and macrophages, respectively. Both cell types were passaged twice a week when they reached 80% confluence. Culture media were supplemented with 10% fetal bovine serum (FBS). Cells were incubated at 37°C with 5% CO_2_.

### Mice bone-marrow-derived neutrophil extraction and infection assays

C57BL/6 female mice (7 to 8 weeks old) were used to obtain bone-marrow-derived neutrophils and for infection assays. Mice were kept in plastic cages in a temperature-controlled environment (22–24°C). Bacteremia was assessed as described elsewhere [30], Briefly, three groups of six female C57BL/6 mice (7 to 8 weeks old) were infected orally with 1×10^5^ colony forming units (CFUs). In parallel, the same number of mice was infected intraperitoneally with 1×10^3^ CFUs of parental or mutant strains in 0.1 ml Phosphate Buffered Saline (PBS) (NaCl 137 mM, KCl 2.7 mM, Na_2_HPO_4_ 10 mM, and K_2_HPO_4_ 1.4 mM, pH 7.4). A group of six females were used as a noninfected control. The health of all mice was monitored daily. After day 3 (for oral infection) or day 5 (for intraperitoneal infection), mice were euthanized by cervical dislocation. Extracted livers and spleens from infected and noninfected controls were weighed and homogenized with sterile PBS; the homogenates were serially diluted (10-fold increments) in sterile PBS, and CFUs were determined by plating onto LB agar. To prepare mouse neutrophils, bone marrow was extracted as described [40], and bone-marrow-derived neutrophils were obtained using the mouse Neutrophil Isolation Kit (Milenybiotec) according to the manufacturer’s instructions. This resulted in the isolation of an average of 800.000 neutrophils/ml with around 85% viability, which were also positive for CD11b and Ly6G, as determined by flow cytometry. In addition, the viability of neutrophils was monitored throughout the experiments with trypan blue staining.

### Gentamicin protection assays

Cell infection assays were conducted using *S*. Typhimurium 14028s and its isogenic derivatives Δ*arcA* and Δ*arcB*, as described [41] with minor modifications. Bacteria were grown under microaerophilic conditions by adding an overlay of 500 μl of sterile mineral oil as a barrier to oxygen. Prior to infection assays, bacteria were centrifuged (13,000 rpm, 5 min) and resuspended in 1 ml of cell culture medium (RPMI for HEp-2, neutrophils and DMEM for RAW 264.7) supplemented with 10% FBS; as a result, the concentration of bacteria used to infect was 5x10^8^ bacteria/ml. For HEp-2 and RAW 264.7 adherent cells, the assays were performed on 96-well plates. 100 μl of the bacterial cell suspension was added to each well containing cell monolayers (multiplicity of infection of 1:100). After 1 h incubation in 5% CO_2_ at 37°C, technical triplicates of the infected cells were stained with trypan blue to determine cell viability. Also, technical replicates for each strain were lysed with deoxycholate (0.5% w/v in PBS), serially diluted (10-fold) in PBS, and plated onto LB agar plates to obtain the CFU of each strain at 1 h postinfection (hpi). The remaining wells were washed twice with sterile PBS and incubated in 5% CO_2_ at 37°C for 2 h with 100 **μl** cell medium plus 250 μg ml^-1^ gentamicin to kill extracellular bacteria. At 3 hpi, the medium was removed, and cells were washed twice with PBS and lysed with sodium deoxycholate (0.5% w/v in PBS). The cell lysates were 10-fold serially diluted in PBS and plated onto LB agar plates to obtained the CFU counts at 3 hpi. The same protocol was used for infection of non-adherent murine neutrophils, except that the cells were kept in 1.5-ml Eppendorf tubes and in each washing step required 5-min centrifugations at 270 *g*.

### Determination of ROS

ROS levels in infected cells were measured using 10 μM of the probe 2',7'-dichlorodihydrofluorescein diacetate (H_2_DCFDA), as described [42]. The fluorescent probe was added just prior to measuring, and fluorescence was determined at 1 and 3 hpi using a TECAN Infinite 200 PRO Nanoquant microplate reader (excitation 480 nm; emission 520 nm). Emission values were first blanked against the background fluorescence of non-infected cells and then normalized to the optical density of bacteria grown as the OD_600_ of the co-culture was measured at the same time as the fluorescence was read. Cells in dimethylsulfoxide (DMSO) were used as a blank. Measurements were carried out every 5 min for 100 min. To calculate intracellular ROS, PBS buffer, bacteria, and eukaryotic cells without treatment were used as blanks. The difference in fluorescence was calculated and divided by the elapsed time. This value was normalized by the difference in growth during the respective times. To quantify H_2_O_2_, the Amplex® Red Hydrogen Peroxide/Peroxidase Assay Kit (ThermoFisher) was used following manufacturer´s instructions. Additionally, HOCl was determined using GFP bleaching. For this, the pGlo plasmid was introduced into each bacterial strain, remaining episomally and induced overnight with arabinose 50 mM [43] and the loss of fluorescence, as an indirect measure of increasing amounts of HOCl, was determined using a TECAN Infinite 200 PRO Nanoquant microplate reader (excitation 395 nm; emission 509 nm).

### Total RNA extraction from infected eukaryotic cells

Infected cells were recovered at 1 and 3 h, washed twice with PBS, and then lysed with sodium deoxycholate (0.5% w/v in PBS). RNA extraction was performed by the acid-phenol method, as described [44]; the pellet was suspended in 30 μl nuclease-free water, and stored at -80°C until used. The integrity of the RNA was determined by 1.0% agarose gel electrophoresis, quantity was determined spectrophotometrically and quality was verified by OD_260_/_280_ ratio. The RNA was treated with 2 U of DNase I (Roche) for 1 h to remove contaminant DNA. To ensure no carry-over DNA in the samples, we routinely performed PCR using primers for bacterial 16s RNA and found no product using the RNA extract as template.

### qRT-PCR

cDNA synthesis was performed at 37°C for 1 h in 25 μl of a mixture containing 2.5 pmol of Random Primers (Invitrogen), 10 μl template RNA (5 mg), 0.2 mM dNTPs, 1 μl sterile water, 4 μl of 5× buffer (250 mM Tris-HCl pH 8.3, 375 mM KCl, 15 mM MgCl2, and 10 mM DTT), and 200 U of reverse transcriptase (Invitrogen). Primers used for qRT-PCR are listed in S1 Table. The relative quantification of each transcript was performed using the Brilliant II SYBR Green QPCR Master Reagent and the Mx3000P detection system (Stratagene). The qRT-PCR mixture (20 μl) contained 1 μl of the cDNA template and 120 nM of each primer. The qRT-PCR was performed under the following conditions: 10 min at 95°C followed by 40 cycles of 30 s at 95°C, 45 s at 58°C, and 30 s at 72°C. Primer pairs were selected with amplification efficiencies of 3.3 ± 10%. Obtained values were used to calculate fold-change expression of target genes, normalized by the expression of a suitable gene selected in these experimental conditions [45]. We used *talB* gene expression for normalization, which is stable under the studied conditions, based on whole transcriptomic analyses [46]. Validation of the *talB* gene expression to normalize transcriptomic experiments under oxidative stress is found in S1, S2 and S3 Figs. The selection was supported by comparing stability and expression of *talB* gene with other commonly used housekeeping genes in our experimental conditions. Expression levels were compared to those of each gene found in strain 14028s at 1 hpi.

### Statistical analyses

Gene expression of each mutant strain was calculated relative to wild type. Gene-by-gene comparisons were performed as individual experiments for each time point using one-way ANOVAs with α *=* 0.05. Statistical analyses were performed with the Bonferroni correction comparing mutant strains with a wild type strain separately at 1 and 3 hpi using GraphPad 5.01 (Prism®).

## Results

### The ArcAB two-component system is required for bacterial survival in epithelial cells, macrophages, and neutrophils

Bacteria associated with epithelial cells (HEp-2), macrophages (RAW 264.7), and bone-marrow-derived neutrophils were quantified at 1 and 3 hpi to evaluate the ability of Δ*arcA* and Δ*arcB* mutants to adhere to cells and to establish an intracellular niche, respectively. Experiments were also performed with the genetically complemented Δ*arcA*::*aph*/pBR::*arcA* and Δ*arcB*::*caf*/pBR::*arcB* strains, which gave similar results to those obtained using the parental strain 14028s.

**Fig 1:**
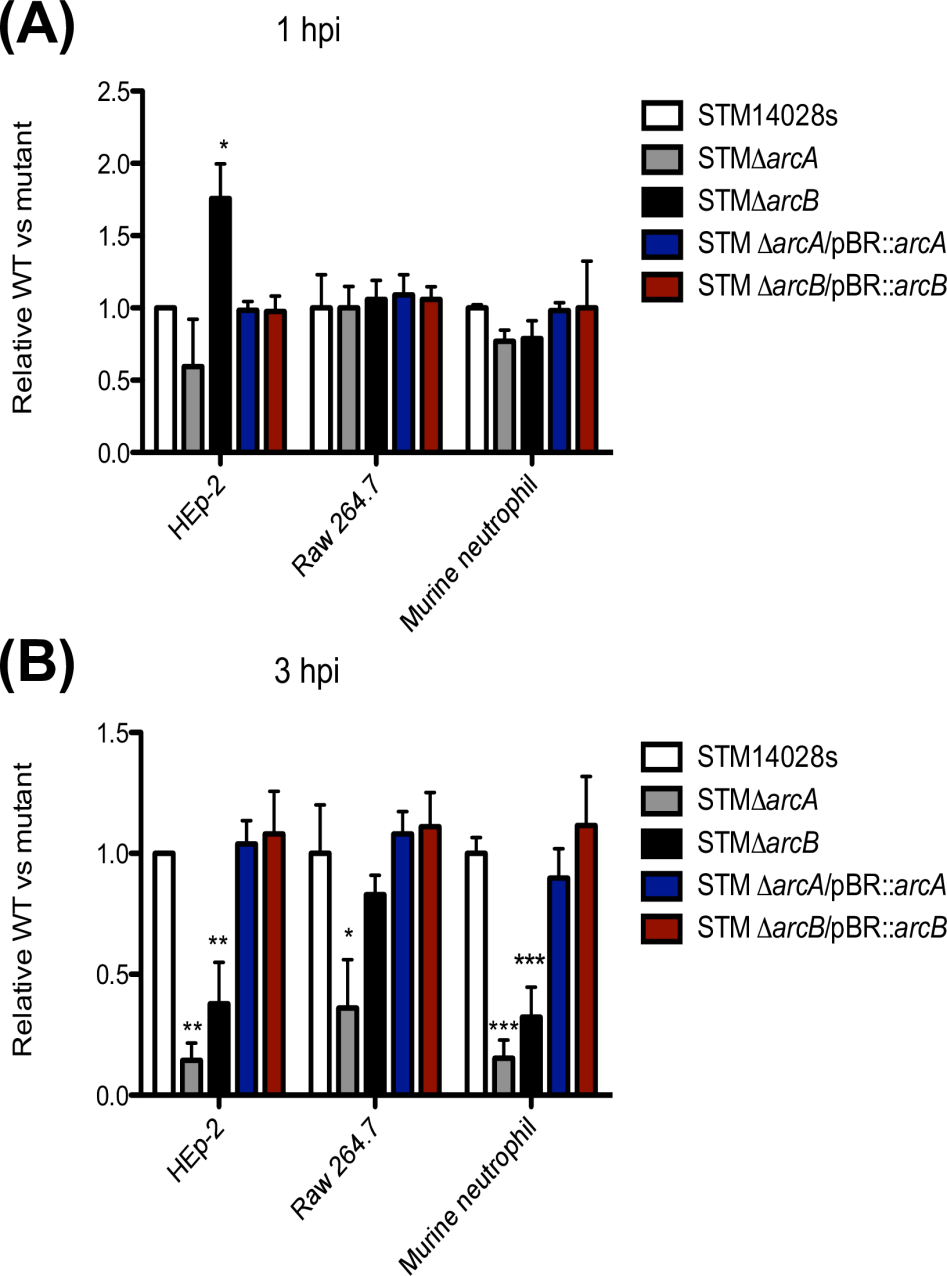
Relative CFUs harvested from mutant vs. wild type *S*. Typhimurium strains. Strains from *S*. Typhimurium were incubated at a MOI of 100 with HEp-2, RAW 264.7 and bone-marrow derived neutrophils. Intracellular bacteria were harvested at 1 hpi (A) and 3 hpi (B) by lysing the cells with sodium deoxycholate. Values are the number of CFU from each strain relative to the number of CFU from the parental strain. Data represent mean ± SD from 5 independent experiments. *P < 0.05; **P < 0.01, ***P < 0.001 by one-way ANOVA followed by Bonferroni *post hoc* test.

*Salmonella* can promote its own entry into host cells by first adhering to the cell surface using specialized fimbriae and adhesins [47, 48]. No difference in adhesion to any of the tested host cells was observed between strains 14028s and Δ*arcA* (Fig 1A), an expected result since ArcA does not regulate genes encoding fimbriae [35], and therefore, its absence should not affect *Salmonella* adhesion. In contrast, loss of ArcB resulted in 2-fold more bacteria harvested from HEp-2 cells, but not from phagocytic cells (Fig 1A). This suggested that absence of the ArcB sensor histidine kinase altered the entry of *Salmonella* into epithelial cells, and this effect was independent from the ArcA response regulator.

At 3 hpi, the number of intracellular bacteria recovered from infections with Δ*arcA* in the various cell types was significantly lower compared to *S*. Typhimurium 14028s (Fig 1B), underscoring the importance of ArcA in establishing an intracellular bacterial niche, especially inside epithelial cells and neutrophils. Similarly, the Δ*arcB* mutant survived poorly in epithelial cells and neutrophils (Fig 1B). As a control, we carried out the same experiment with a previous incubation of the eukaryotic cells with a phagocytosis inhibitor and found that this treatment reduced drastically the number of recovered CFUs (S4 Fig).

**Fig 2:**
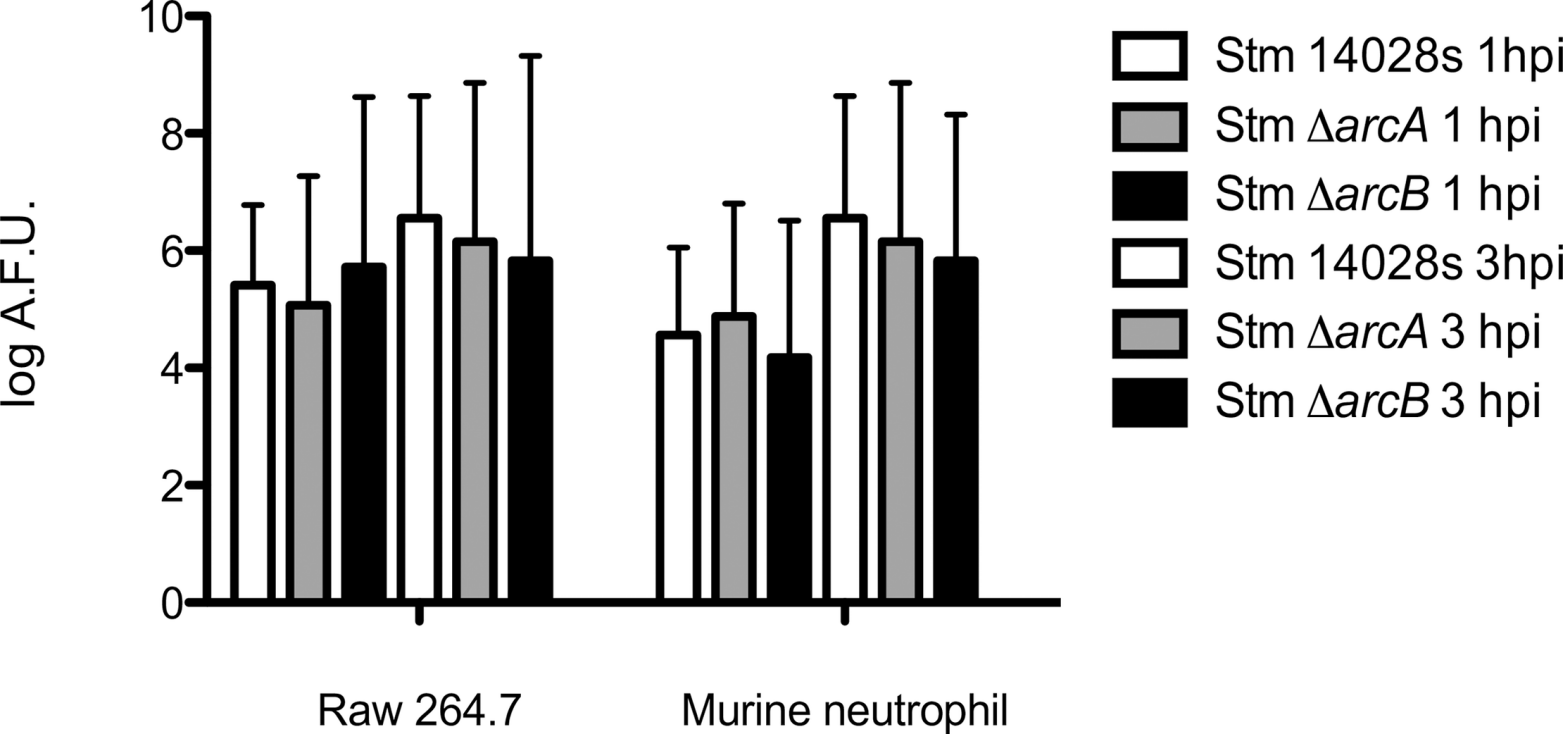
Total reactive oxygen species production. *S*. Typhimurium strains were incubated at a MOI of 100 with each cell type and ROS were evaluated at 1 and 3 hpi. The amount of ROS was determined by quantifying DCF fluorescence in neutrophils and macrophages co-cultured with the parental *S*. Typhimurium 14028s, and the Δ*arcA* and Δ*arcB* mutants. Values indicate Arbitrary Fluoresce Units (AFU) normalized by the number of bacteria recovered. One-way ANOVA followed by Bonferroni *post hoc* test.; no significant differences found at α *=* 0.05. Biological replicates *n* = 5, three technical replicates per experiment.

The reduced intracellular survival of Δ*arcA* and Δ*arcB* mutants (Fig 1) could be due to either increased bacterial susceptibility to intracellular microbicidal effects or a differential induction of ROS upon infection. To distinguish between the two possibilities, we determined the amount of ROS produced by infected cells at the same time points postinfection using H_2_DCFDA. This is a fluorogenic probe that in the presence of ROS is converted to the highly florescent compound 2',7'-dichlorofluorescein (DCF). The probe detected similar amounts of ROS over 3 hpi (Fig 2), which remained relatively constant irrespective of the infecting bacterial strain in the assay. This suggested that both macrophages and neutrophils maintain their total ROS levels throughout the first 3 h of infection irrespective of the presence or absence of *arcAB* gene expression. Similar results were observed with the quantification of H_2_O_2_ production in both cell types and HOCl production in neutrophils (S5 and S6 Figs, respectively). Together, we conclude that an intact ArcAB two-component system contributes to intracellular survival of *S*. Typhimurium in phagocytes and epithelial cells, and the presence or absence of the system does not influence the levels of ROS production induced in host cells.

### ArcA and ArcB regulate detoxification-, membrane permeability-, and toxic resistance-related genes inside macrophages and neutrophils

We evaluated the role of the ArcAB two-component system in modulating oxidative stress resistance during intracellular bacterial survival [17]. RNA was extracted from bacteria after infection (RAW 264.7 and bone-marrow-derived neutrophils) at 1 and 3 hpi. These times were representative of two stages in the eukaryotic cell response, namely the oxidative burst and SCV maturation, respectively [49–53].

**Fig 3:**
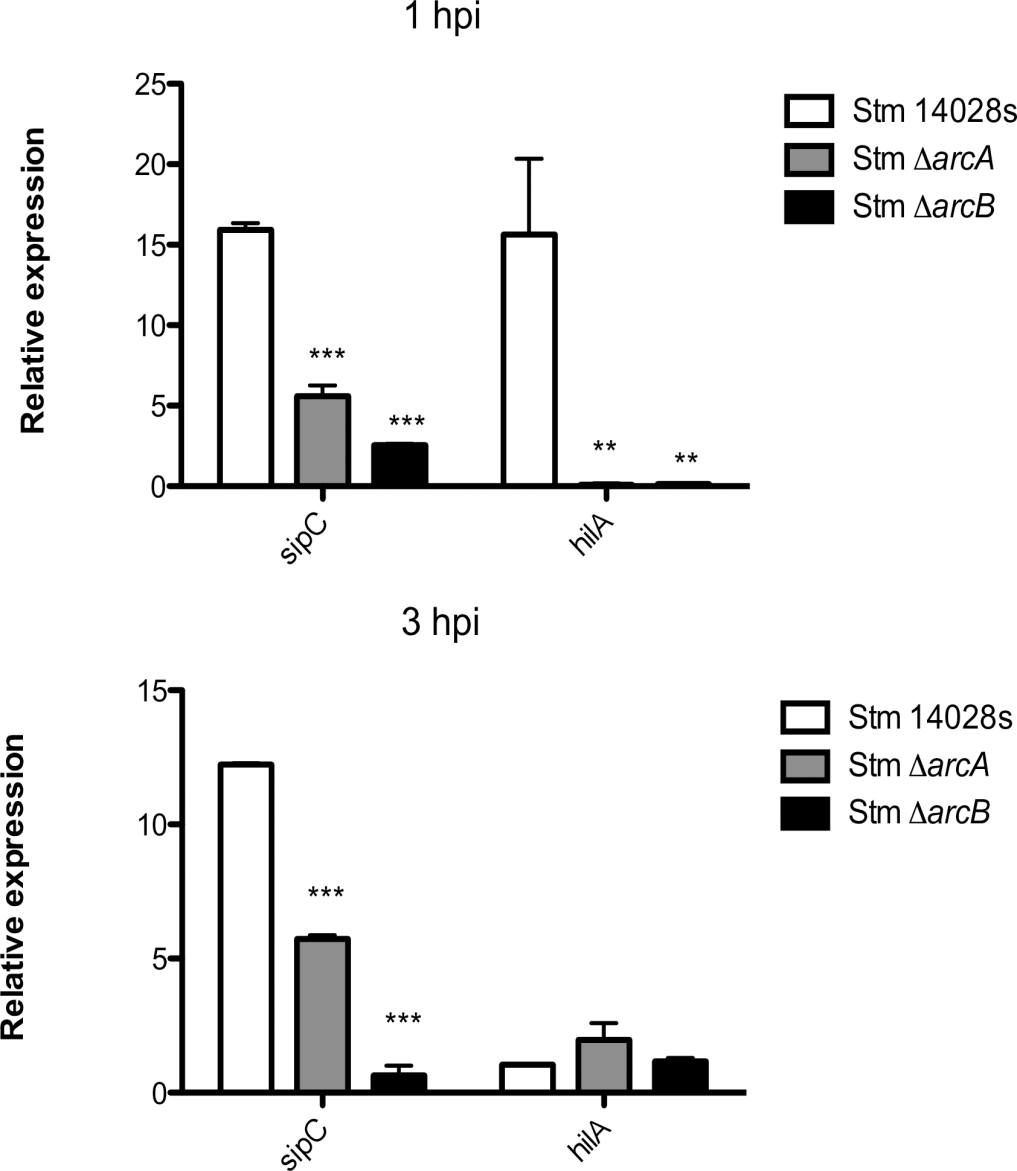
Relative expression of SPI-1 genes *sipC* and *hilA* in *S*. Typhimurium Δ*arcA* and Δ*arcB* strains inside bone-marrow-derived murine neutrophils. The effect of Δ*arcA* and Δ*arcB* on SPI-1 genes *sipC* and *hilA* was determined from bacteria isolated from infected bone-marrow-derived murine neutrophils. *P < 0.05; **P < 0.01; ***P < 0.001. One-way ANOVA with the Bonferroni correction comparing mutant strains with a wild type strain separately at 1 and 3 h postinfection. Biological replicates *n* = 5, three technical replicates per experiment.

It was previously demonstrated that ArcA and Hfq mediate the activation of the *hilD* promoter, especially during early stationary phase and with vigorous aeration [54]. This can trigger activation of SPI-1 resulting in the secretion of effector proteins required for *Salmonella* uptake by cells [54]. We therefore examined the expression of *hilA* and *sipC*, as these genes are important during the early steps of *Salmonella* intracellular survival. In neutrophils, ArcA was required for induction of *sipC* expression (Fig 3). SipC is a protein whose function contributes to the maintenance of the phagosome compartment [54]. Also, *hilA* expression was upregulated by ArcA at 3 hpi under our experimental conditions (Fig 3). In early stationary phase *in vitro* culture, mutations in *arcA* also reduced *hilA* expression 2-fold [54].

**Fig 4:**
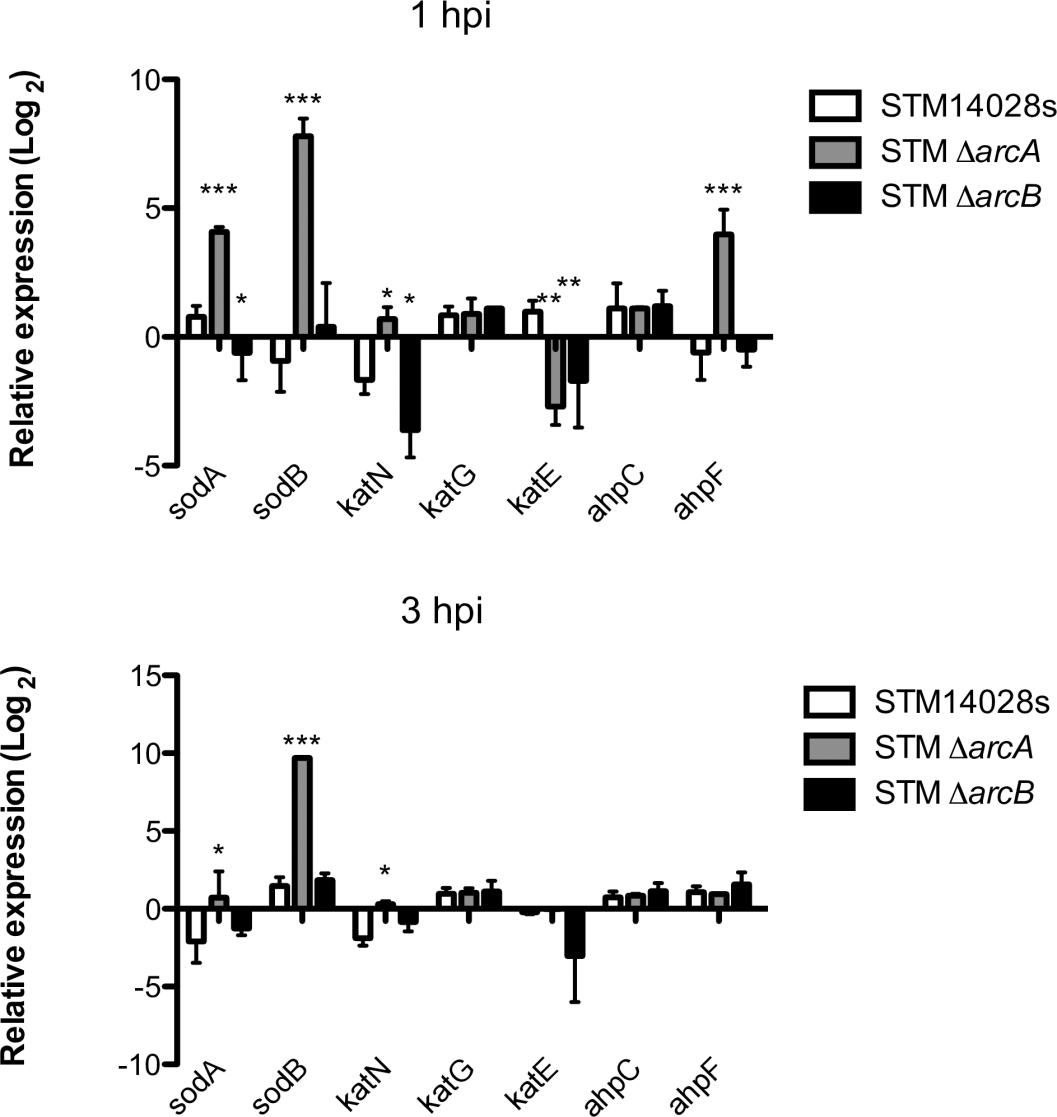
Relative expressions of genes related to detoxification (*sodA*, *sodB*, *katN*, *katG*, *katE*, *ahpC* and *ahpF*) in *S*. Typhimurium Δ*arcA* and Δ*arcB* strains inside bone-marrow-derived murine neutrophils. *P < 0.05; **P < 0.01; ***P < 0.001. One-way ANOVA with the Bonferroni correction comparing mutant strains with a wild type strain separately at 1 and 3 h postinfection. Biological replicates *n* = 5, three technical replicates per experiment.

Other important factors required for *Salmonella* survival inside phagocytes are detoxification enzymes including *katG*, *katE*, *katN*, *ahpF*, and *ahpC*, which become highly induced in response to hydrogen peroxide [17]. Inside neutrophils, where the main ROS compound is HOCl, the majority genes encoding catalases, alkyl hydroperoxidase, and superoxide dismutase were up-regulated in the absence of *arcA* (Fig 4). In the context of biochemical reactions occurring inside this particular phagosome, where superoxide and hydrogen peroxide are consumed rapidly to produce HOCl in the presence of the myeloperoxidase enzyme, these products are arguably scarce and short-lived. Only *katE* was down-regulated in the absence of *arcA* at 1 hpi (Fig 4). Because the KatE enzyme is expressed during exponential growth [55], it is likely that this result reflects the reduced intracellular growth of *arcA* mutant bacteria.

**Fig 5:**
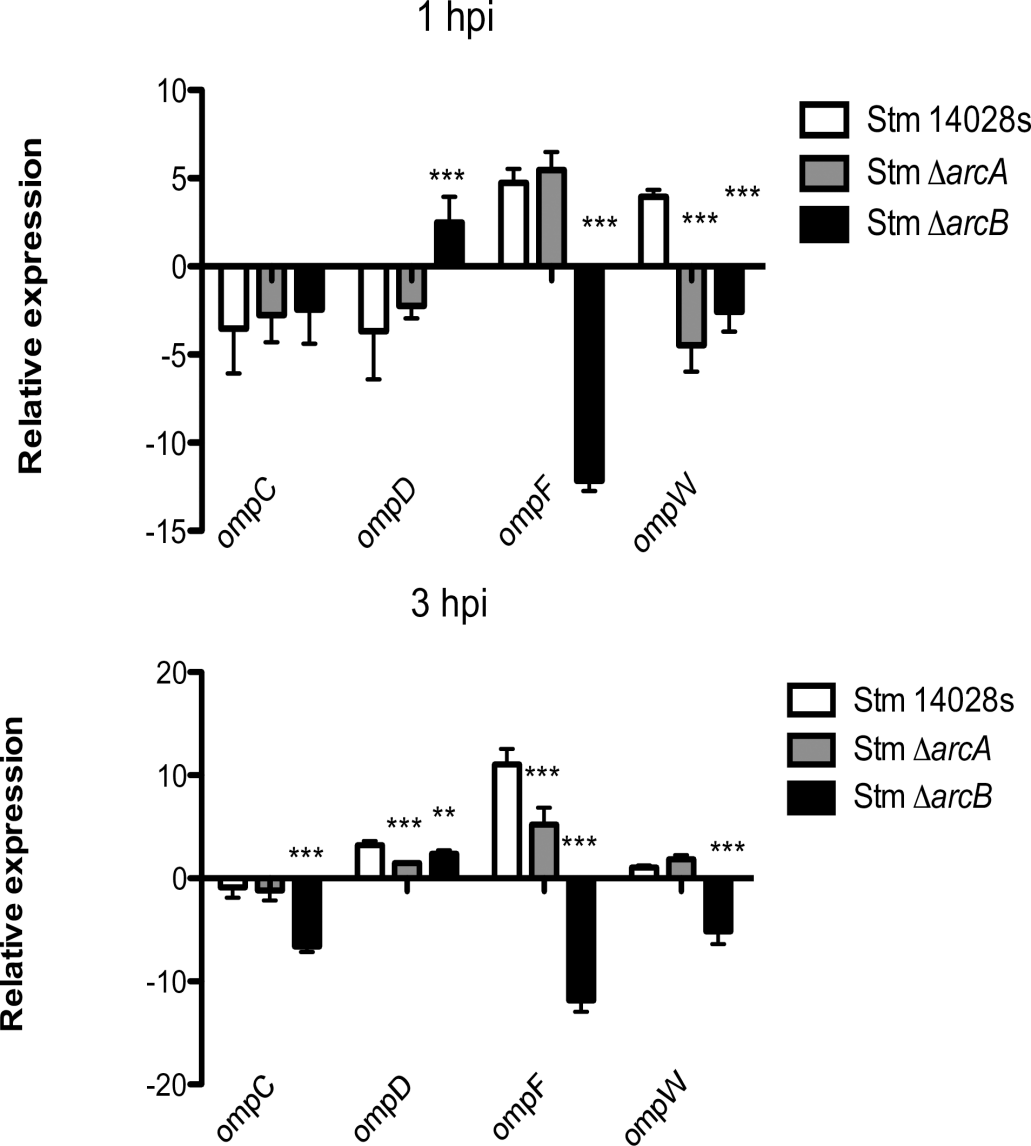
Relative expression of porin genes (*ompC*, *ompD*, *ompF* and *ompW*) in *S*. Typhimurium Δ*arcA* and Δ*arcB* strains inside bone-marrow-derived murine neutrophils. Δ*arcA* and Δ*arcB* strains inside bone-marrow-derived murine neutrophils. *P < 0 05; **P < 0 01; ***P < 0 001. One-way ANOVA with the Bonferroni correction comparing mutant strains with a wild type strain separately at 1 and 3 h postinfection. Biological replicates *n* = 5, three technical replicates per experiment.

Another bacterial protection mechanism against ROS involves the modulation of the composition of the outer membrane. Under H_2_O_2_ and HOCl stress *in vitro*, *S*. Typhimurium ArcA downregulates genes encoding OmpC, OmpD, OmpF, and OmpW porins [28, 29]. Inside neutrophils, however, bacteria through ArcA upregulate *ompD*, *ompF*, and *ompW* (Fig 5), while in macrophages ArcA downregulates *ompD* and *ompF* and upregulates *ompW* (S7 Fig). These results showing different expression patterns related to the presence or absence of *arcA* and the type of phagocytic cell suggest that bacteria sense different environments and respond accordingly in each cell type, this adaptation capacity allow *Salmonella* to survive the ROS-related conditions that the bacteria encounters during infection.

**Fig 6:**
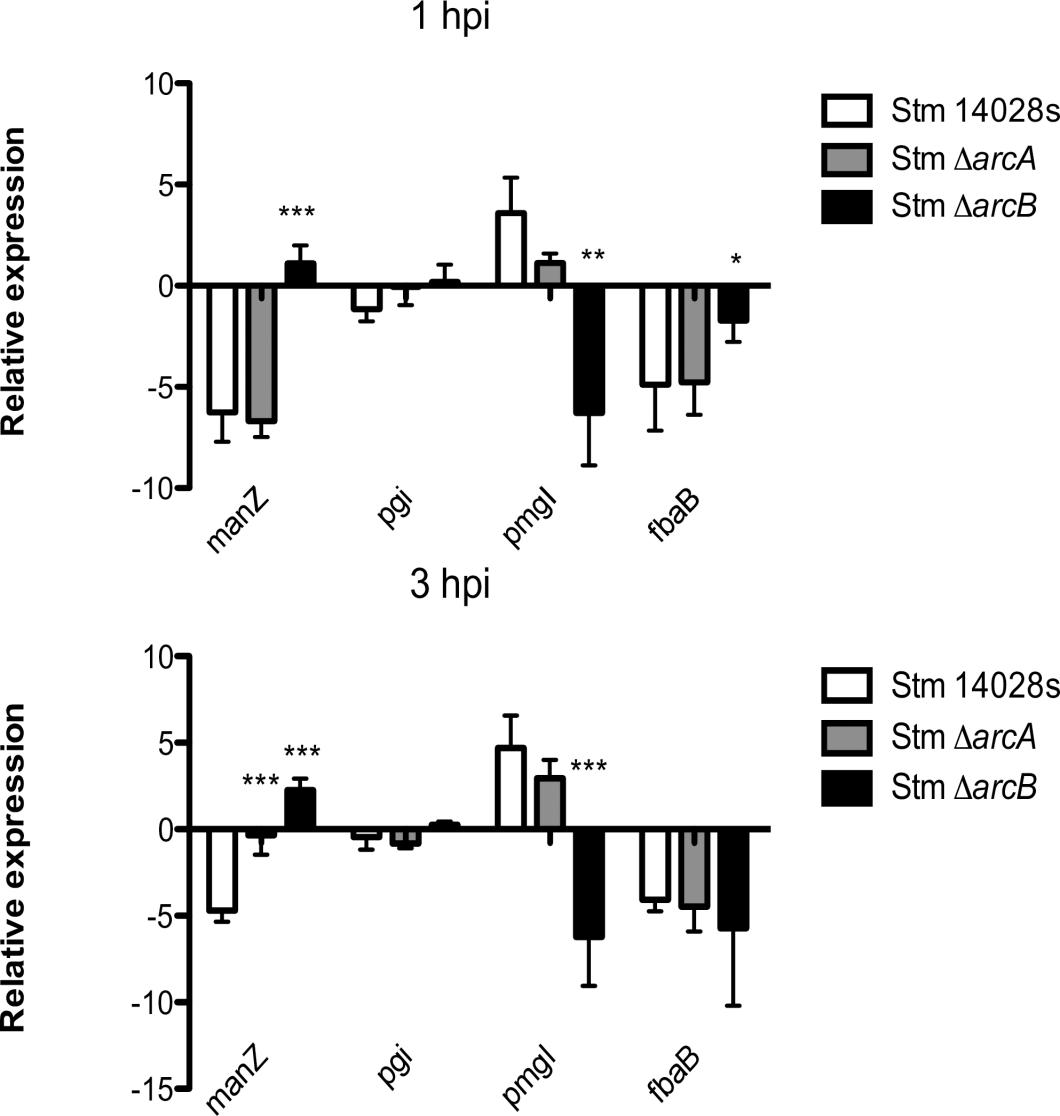
Relative expression of metabolic genes (*manZ*, *pgi*, *pmgI* and *fbaB*) in *S*. Typhimurium Δ*arcA* and Δ*arcB* strains inside bone-marrow-derived murine neutrophils. Δ*arcA* and Δ*arcB* strains inside bone-marrow-derived murine neutrophils. *P < 0.05; **P < 0.01; ***P < 0.001. One-way ANOVA with the Bonferroni correction comparing mutant strains with a wild type strain separately at 1 and 3 hours postinfection. Biological replicates *n* = 5, three technical replicates per experiment.

Gene expression related to central metabolism in response to highly toxic HOCl was also evaluated since these pathways are critical to maintain redox state and prevent oxidative damage, and previous work reported that ArcA, in response to 1 mM H_2_O_2_, regulates key genes required for carbon biosynthesis, energy, and maintenance of the redox state [35] as the main bacterial response to these toxic conditions. We examined the expression of a selection of genes encoding enzymes involved in central metabolism. These included: *manZ* (encoding a subunit from the mannose transporter) and three genes encoding enzymes involved in glycolysis, namely phosphoglucose isomerase (*pgi*), 2,3-bisphosphoglycerate-independent phosphoglycerate mutase (*pmgI*), and fructose-bisphosphate aldolase (*fbaB*). Our results show that genes related to glycolysis are not part of the ArcA regulon. In contrast, *manZ* is downregulated by ArcA at 3 hpi (Fig 6). A similar finding was also reported in *E*. *coli*, where complex posttranslational regulation of this transporter in response to low glucose and phosphate was noted [56]. We also found a similar response in macrophages (S8 Fig).

### *arcA* and *arcB* are required for successful systemic infection in C57BL/6 mice

**Fig 7:**
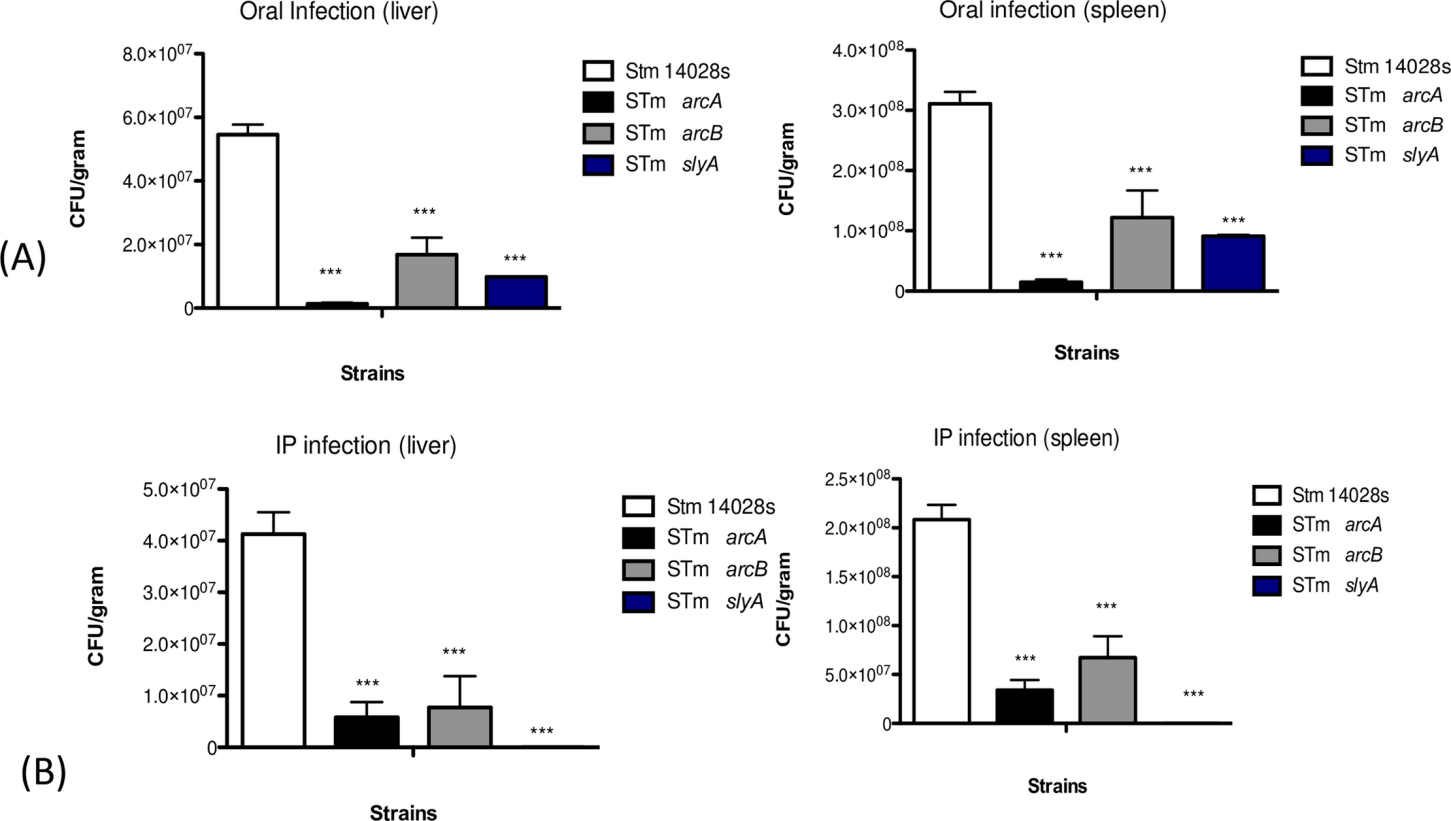
Recovery of *Salmonella* in C57BL/6 mice. (A) Mice orally infected with 1×10^5^ bacteria/100 μl *S*. Typhimurium 14028s. (B) Mice intraperitoneally infected with 1×10^3^ bacteria/100 μl *S*. Typhimurium 14028s. Δ*arc*A and Δ*arc*B strains were grown until OD_600_ = 0.2 in microaerophilic conditions. One-way ANOVA with the Bonferroni correction. *P < 0.05; **P < 0.01, ***P < 0.001. Biological replicates *n* = 5, five technical replicates per experiment.

To investigate the relevance of *arcA* and *arcB* during host infection, *in vivo* infection assays were conducted in C57BL/6 mice with each single mutant. In comparison to strain 14028s, both Δ*arcA* and Δ*arcB* showed reduced abilities to cause infection when administered orally or intraperitoneally. Upon oral administration of 1×10^5^ CFU, the recovery of CFUs corresponding to the Δ*arcA* strain was reduced more than 90% in both the liver and spleen. Likewise, Δ*arcB* diminished approximately 60–70% in both organs compared to the wild type strain (Fig 7). A similar result was observed after intraperitoneal infection with a 1×10^3^-CFU inoculum. Recovery of the Δ*arcA* strain was reduced 85% in the liver and 83% in the spleen. The same phenomenon was observed when quantifying viable bacteria from the Δ*arcB* strain (Fig 7), demonstrating that ArcA and ArcB are essential for *S*. Typhimurium systemic infection in C57BL/6 mice separately, as seen in the CFU recovered from each singe mutant. As a control, we used a Δ*slyA* strain, which is highly attenuated in mice infections [57, 58]. Inactivating SlyA decreased the recovery of bacteria in a manner similar to that observed Δ*arcA* and Δ*arcB* strains. These results, combined with the gene expression patterns observed for each single mutant, underscore the importance of ArcA and ArcB for systemic infection.

## Discussion

ArcA regulates *Salmonella* cellular metabolism, flagella biosynthesis, and motility under many *in vitro* conditions, including anaerobiosis, aerobiosis, and the presence of H_2_O_2_ [34, 35]. Loss of *arcA* also reduces expression of SPI-1 genes *invF* and *hilA* in liquid cultures [54]. However, the involvement of ArcA in *Salmonella* intracellular survival remains unclear. In this study, we investigated the involvement of ArcA in cell invasion and survival in epithelial cells, macrophages, and neutrophils in physiological conditions similar to those found in the host. Our results revealed that ArcA plays a role in intracellular bacterial survival, as demonstrated by the significant reduction in the number of viable Δ*arcA* bacteria recovered from each cell type at 3 hpi. The reduced numbers of bacteria were related to their reduced capacity to replicate within cells and not to defects in their ability for invasion/phagocytosis. Significant differences were not observed in the overall production of ROS by infected macrophages and neutrophils, irrespective of the genetic background of the infecting bacterial strain. This observation suggests that the cues triggering ROS production are independent of the ArcAB regulon. However, the bacteria were in an oxidative environment when survival was measured, which was consistent with the peak in ROS production by macrophages and neutrophils upon infection [59–61].

We found that at the transcriptional level, ArcA modulates adaptive bacterial responses to stress conditions inside phagocytes. In neutrophils, which display a harsher environment than macrophages, ArcA was implicated in promoting the expression of genes associated with the SPI-1 T3SS such as *hilA* and *sipC*, both of which are associated with invasion, as well as formation and maintenance of the SCV [54, 62]. Additionally, inside neutrophils, only some of the typical genes that encode detoxifying enzymes were induced, as previously described for macrophages [17]. Of these enzymes, only *katE* seems to be relevant in the conditions encountered inside neutrophils during the time range evaluated.

The expression of porin genes was also modulated by ArcA in response to several factors found in the context of infection. For instance, OmpC and OmpF also respond to osmolarity and in this case are regulated by EnvZ and OmpR [63]; also permeability varies with glucose levels, including OmpW [64, 65], while OmpD is modulated by oxygen levels and changing pH values [66]. Additionally, the glycolytic pathway was independent of the ArcA regulon, although it seems to be required for intracellular replication of *S*. Typhimurium in macrophages [67]. Only the endpoint of the pathway was induced by ArcA in this case, which could be also related to the interconnection between glycolysis and the pentose phosphate pathway.

In the systemic infection model, significantly reduced numbers of viable bacteria were found in spleen and liver, suggesting ArcAB play an important role in the *in vivo* infection ability of *S*. Typhimurium. This observation agrees with our results from *ex vivo* cell infections and also with our transcriptional analyses. These results differ from the previous report by Evans et al. where no differences in virulence were found for *arcA* and wild type *S*. Typhimurium [34]. These could be due to methodological variations in the infection assays. In the report by Evans et al., a lower bacterial inoculum (250 CFU per mice) was used and the mice remained alive for several days. In contrast, a higher bacterial dose, as we have used, would cause a more robust level of inflammation by the wild type bacteria leading to rapid killing of the infected mice, while the *arcA* and *arcB* mutants are clearly attenuated. We believe our experimental conditions model the infective dose for *Salmonella* under natural infections, which is high (10^4^−10^8^) in comparison to other pathogens [68]. Intestinal invasion of *S*. Typhimurium depends on the bacterial dose and a linear proportion between the inocula (10^4^−10^10^) and invasion has been shown [69]. These observations were also replicated in the wax moth larvae infection model [70].

Evans et al. also reported no differences in virulence between *arcA* mutant and parental strains using a competitive index (CI) assay that included high bacterial doses [34], results we also confirmed in our study (S9 Fig). The absence of an effect of *arcA* and *arcB* mutants in competition assays at low bacterial doses can be explained by the dose effect discussed above. Under higher doses, we believe the parental strain in the mixed infection can elicit a robust inflammatory response, which would preclude seeing differences with the mutants. Together, we conclude that the ArcAB regulon, due to its modulatory role in central metabolism, fine tunes the expression of several stress-associated pathways having a more noticeable role under conditions of higher bacterial cell density.

The expression analysis of selected genes shows that the ArcAB system may work in a non-cognate manner, since there is a different gene expression pattern in each single mutant strain (Figs 4, 5 and 6). This has been previously observed by others under different experimental conditions [71–75], and also noticed in our global transcriptomic analyses in the presence of H_2_O_2_ and HOCl (in preparation). It is possible that ArcAB may be noncanonically activated under certain metabolic conditions. For example, intracellular infection could increase the pool of acetyl phosphates, which would activate ArcA independently of ArcB phosphorylation. Global transcriptomic analyses under multiple stress conditions including intracellular infection may offer clues to understand this behavior of the ArcAB system.

## Supporting information

S1 Fig Variation of 16S under oxidative stress. Kegg Pathway illustration of data obtained in whole transcriptome analyses of the parental strain *S*. Typhimurium 14028s. Data extracted from RNA-seq analyses of each strain under 1.56 mM of H_2_O_2_ and 1.0075 mM of NaOCl separately. Green boxes indicate repression; red boxes indicate induction of the particular gene. The expression of the 16S gene increases in the presence of H_2_O_2_ (A) and decreases in the presence of NaOCl (B). The raw data is deposited in the NCBI SRA database under accession numbers SRR5192881 and SRR5192882 (Bioproject PRJNA357075) [46].
(TIF)

S2 Fig Stability of tested housekeeping genes. The abundance of transcripts of 16S, *talB*, *gyrB*, *rpoB*, *ftsZ*, *secA*, *gmk*, and *glnA* under hydrogen peroxide (H_2_O_2_) and sodium hypochlorite (NaOCl), as FPKM (Fragments per kilo base per million [mapped reads]) values, was used to calculated fold change expression between the conditions: under 1.56 mM of H_2_O_2_ (grey bar), under 1.0075 mM of NaOCl (yellow bar) and Control (blue bar). FPKM represents the normalized abundance of transcripts values in a particular condition.
(TIF)

S3 Fig Expression of target genes calculated as relative to different housekeeping genes. Comparison between the expression patterns of some of our work target genes (*manZ*, *pmgI*, *ompD*, *ompW*, *sodA*, and *sipC*) in control, H_2_O_2_ and NaOCl conditions, normalized with some of the housekeeping genes proposed by Rocha et al., 2015 and *talB*. Ratios were calculated using the FPKM values for all genes in each condition as a measure of expression. Fold change expression of the genes are shown under Control (blue bar), 1.56 mM of H_2_O_2_ (orange bar) and 1.0075 mM of NaOCl (grey bar) conditions.
(TIF)

S4 Fig CFUs recovered from eukaryotic cells treated with 5 μM cytochalasin D. Strains from *S*. Typhimurium were incubated at a MOI of 100 with (A) macrophages and (B) bone-marrow derived neutrophils and CFU was recovered as indicated in Materials and Methods in gentamicin protection assays. Values represent Colony Forming Units (CFU) recovered of each strain infecting the phagocytes and expressed as the Ratio of CFU recovered from phagocytes (macrophages and neutrophils) treated vs un treated with cytochalasin D. *S*. Typhimurium 14028s harvested from cells untreated phagocytes (black bar), *S*. Typhimurium 14028s harvested from treated phagocytes (white bar), *S*. Typhimurium Δ*arcA* harvested from untreated phagocytes (dark blue bar), *S*. Typhimurium Δ*arcA* harvested from treated phagocytes (light blue bar), *S*. Typhimurium Δ*arcB* from untreated phagocytes (dark grey bar), and *S*. Typhimurium Δ*arcB* from treated phagocytes (light grey bar) at 3 hpi. *P < 0.05; **P < 0.01, ***P < 0.001 by one-way ANOVA followed by Bonferroni *post hoc* test.
(TIF)

S5 Fig H_2_O_2_ production in Raw 264.7 and bone-marrow-derived murine neutrophils. Phagocytic cells were co-cultured with *S*. Typhimurium 14028s (white bar), *S*. Typhimurium Δ*arcA* (grey bar) and *S*. Typhimurium Δ*arcB* (black bar) at 1 hpi, and *S*. Typhimurium 14028s (blue bar), *S*. Typhimurium Δ*arcA* (red bar), and *S*. Typhimurium Δ*arcB* (purple bar) at 3 hpi. Quantified using Amplex® Red Hydrogen Peroxide/Peroxidase Assay Kit. One-way ANOVA followed by Bonferroni *post hoc* test., no significate difference found. Data are from 5 biological replicates with 3 technical replicates per experiment.
(TIF)

S6 Fig HOCl production in bone-marrow-derived murine neutrophils. Each phagocyte was co-cultured with *S*. Typhimurium 14028s/pGLO (white bar), *S*. Typhimurium Δ*arcA/*pGLO (grey bar), and *S*. Typhimurium Δ*arcB/*pGLO (black bar). The amount of HOCl was quantified by GFP bleaching after cell lysate. *P < 0.05; **P < 0.01, ***P < 0.001 by one-way ANOVA followed by Bonferroni *post hoc* test. Biological replicates n = 5, 3 technical replicates in each one. Data are from 5 biological replicates with 3 technical replicates per experiment.
(TIF)

S7 Fig Relative expression of genes in the *S*. Typhimurium 14028s, Δ*arcA*, and Δ*arcB* strains inside RAW 264.7 cells. *p<0,05; **p<0,01; ***p<0,001. One Way ANOVA with Bonferroni post-test comparing mutant strains *vs* wild type strain in 1 hpi and 3 hpi separately. Data are from 5 biological replicates with 3 technical replicates per experiment.
(TIF)

S8 Fig Relative expression of genes in the *S*. Typhimurium 14028s, Δ*arcA*, and Δ*arcB* strains inside RAW 264.7 cells. *p<0,05; **p<0,01; ***p<0,001. One Way ANOVA with Bonferroni post-test comparing mutant strains vs wild type strain in 1 hpi and 3 hpi separately. Data are from 5 biological replicates with 3 technical replicates per experiment.
(TIF)

S9 Fig Competitive infection assays. Competitive infections were performed as described before by Evans et al., 2011 [34]. Animals where infected orally (p.o.) of i.p with a 1:1 mixture of S. Typhimurium 14028 and the *arcA* mutant. Mice were sacrificed at 4 or 6 days p.i and liver and spleen were collected for processing. CI index was calculated as described [34].
(TIF)

S1 Table Primers used for qPCR. (PDF)
