# Correction: The ArcAB two-component regulatory system promotes resistance to reactive oxygen species and systemic infection by *Salmonella* Typhimurium

**DOI:** 10.1371/journal.pone.0214634

**Published:** 2019-03-26

**Authors:** Coral Pardo-Esté, Alejandro A. Hidalgo, Camila Aguirre, Ana Inostroza, Alan C. Briones, Carolina E. Cabezas, Juan Castro-Severyn, Juan A. Fuentes, Cecilia M. Opazo, Claudia A. Riedel, Carolina Otero, Rodrigo Pacheco, Miguel A. Valvano, Claudia P. Saavedra

Dr. Ana Inostroza should be included in the author byline. They should be listed as the fourth author, and their affiliation is 1: Laboratorio de Microbiología Molecular, Departamento de Ciencias Biológicas, Facultad de Ciencias de la Vida, Universidad Andres Bello, Santiago, Chile. The contributions of this author are as follows: acquisition of data, analysis and interpretation of data, the development of methodology, and making a critical review.

The correct citation is: Pardo-Esté C, Hidalgo AA, Aguirre C, Inostroza A, Briones AC, Cabezas CE, et al. (2018) The ArcAB two-component regulatory system promotes resistance to reactive oxygen species and systemic infection by *Salmonella* Typhimurium. PLoS ONE 13(9): e0203497. https://doi.org/10.1371/journal.pone.0203497
