# Supplementary figures and images for: The ArcAB two-component regulatory system promotes resistance to reactive oxygen species and systemic infection by Salmonella Typhimurium
Source: PLoS One. 2018 Sep 4;13(9):e0203497. doi: 10.1371/journal.pone.0203497 (PMC6122832; doi:10.1371/journal.pone.0203497)

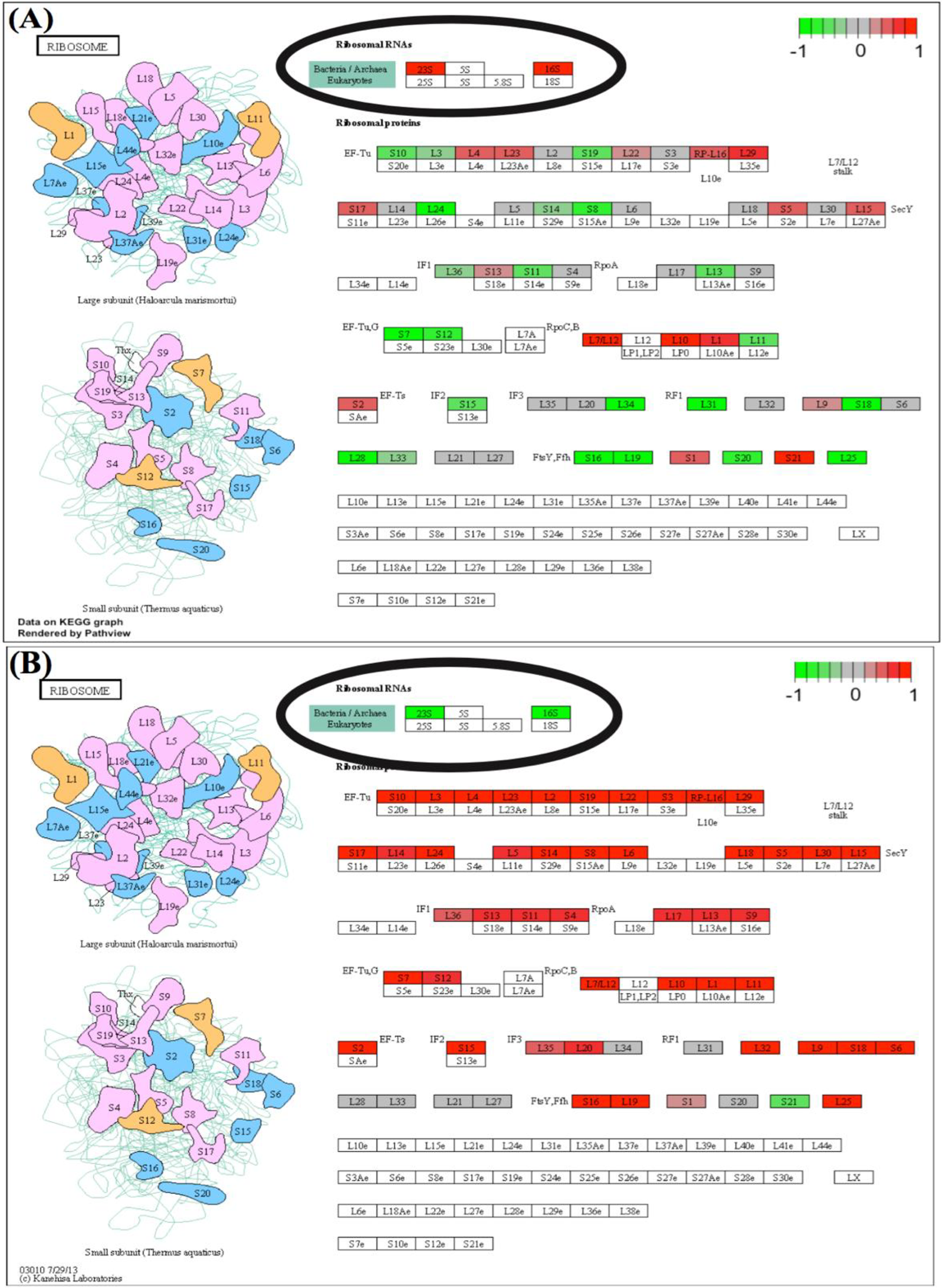

Supplement: S1 Fig — Kegg Pathway illustration of data obtained in whole transcriptome analyses of the parental strain S. Typhimurium 14028s. Data extracted from RNA-seq analyses of each strain under 1.56 mM of H2O2 and 1.0075 mM of NaOCl separately. Green boxes indicate repression; red boxes indicate induction of the particular gene. The expression of the 16S gene increases in the presence of H2O2 (A) and decreases in the presence of NaOCl (B). The raw data is deposited in the NCBI SRA database under accession numbers SRR5192881 and SRR5192882 (Bioproject PRJNA357075) [46]. (TIF) [file pone.0203497.s001.tif]

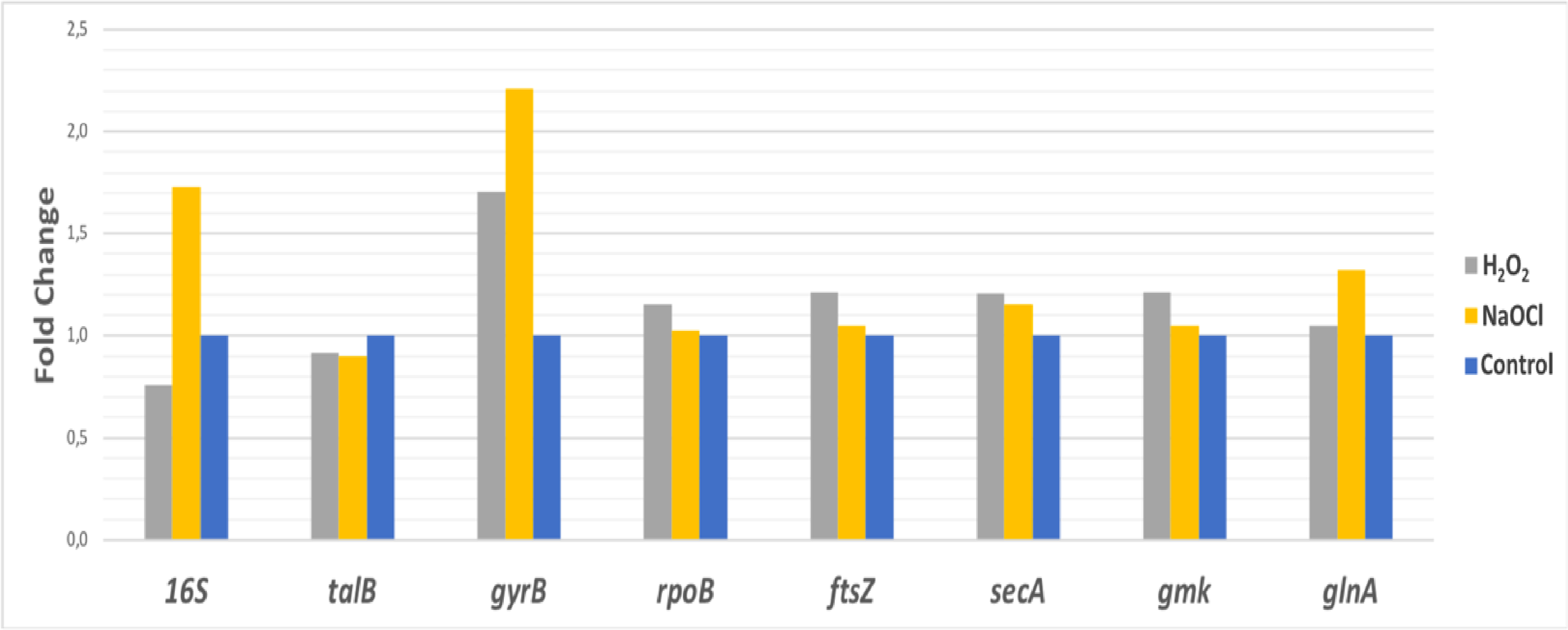

Supplement: S2 Fig — The abundance of transcripts of 16S, talB, gyrB, rpoB, ftsZ, secA, gmk, and glnA under hydrogen peroxide (H2O2) and sodium hypochlorite (NaOCl), as FPKM (Fragments per kilo base per million [mapped reads]) values, was used to calculated fold change expression between the conditions: under 1.56 mM of H2O2 (grey bar), under 1.0075 mM of NaOCl (yellow bar) and Control (blue bar). FPKM represents the normalized abundance of transcripts values in a particular condition. (TIF) [file pone.0203497.s002.tif]

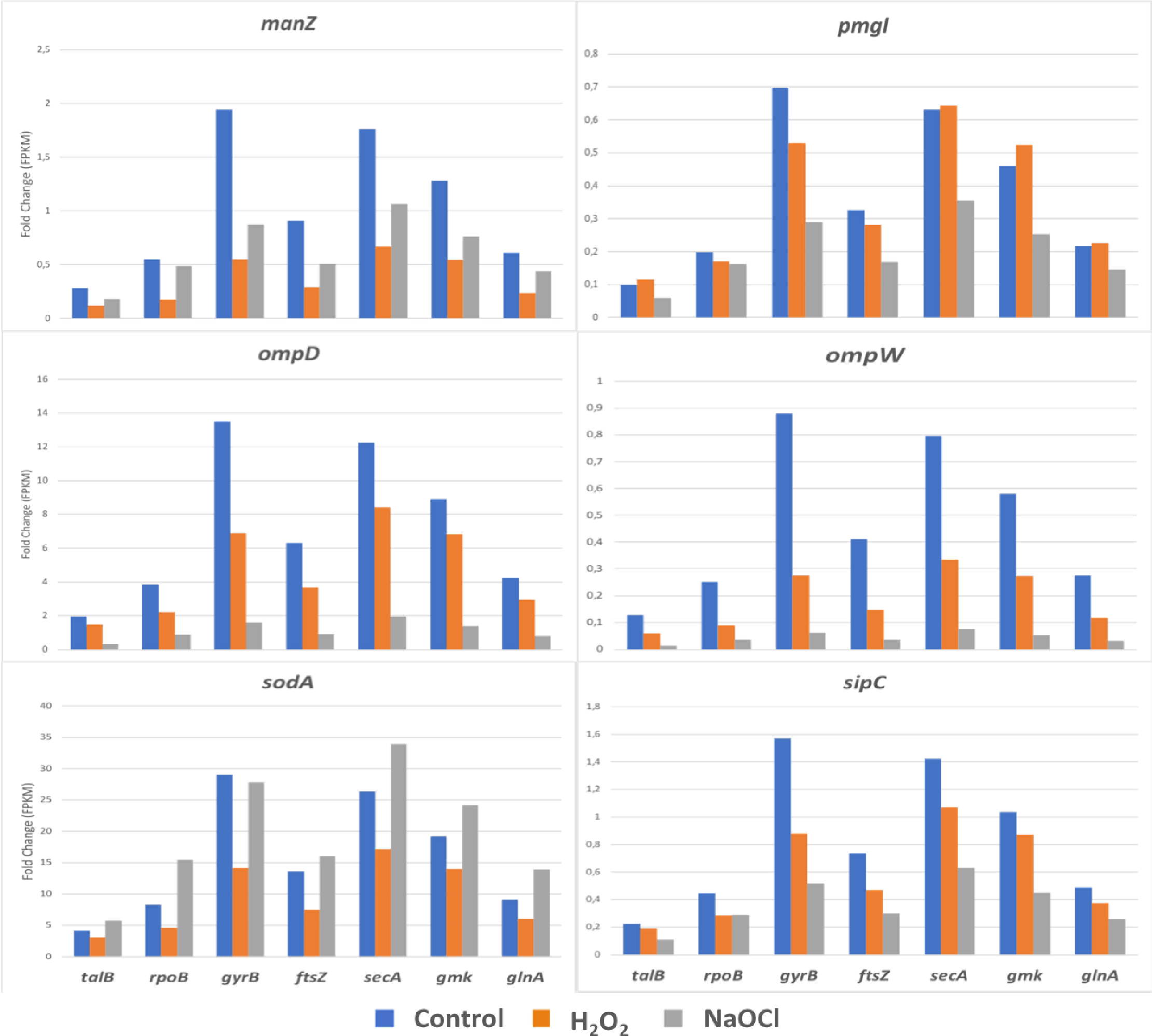

Supplement: S3 Fig — Comparison between the expression patterns of some of our work target genes (manZ, pmgI, ompD, ompW, sodA, and sipC) in control, H2O2 and NaOCl conditions, normalized with some of the housekeeping genes proposed by Rocha et al., 2015 and talB. Ratios were calculated using the FPKM values for all genes in each condition as a measure of expression. Fold change expression of the genes are shown under Control (blue bar), 1.56 mM of H2O2 (orange bar) and 1.0075 mM of NaOCl (grey bar) conditions. (TIF) [file pone.0203497.s003.tif]

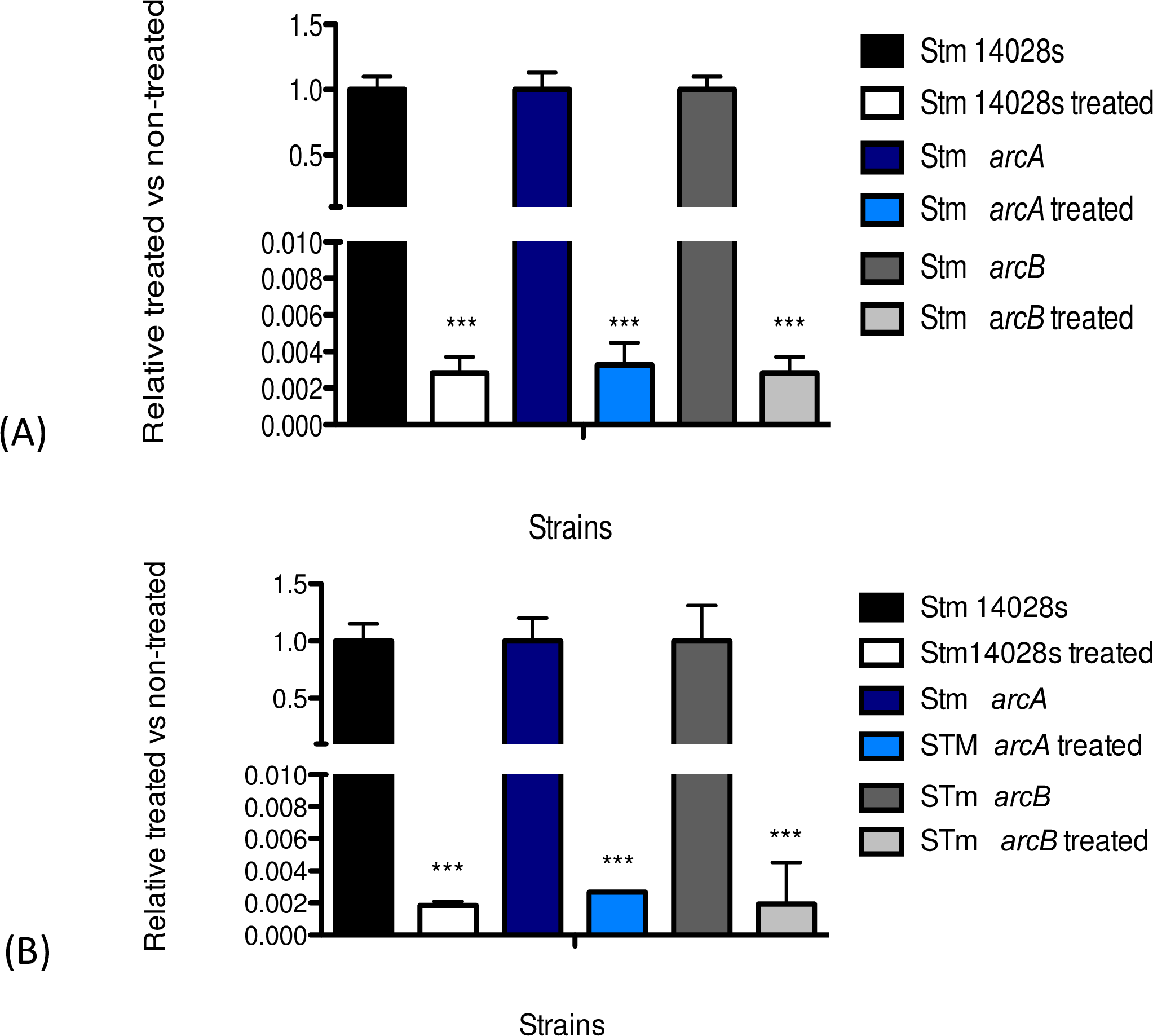

Supplement: S4 Fig — Strains from S. Typhimurium were incubated at a MOI of 100 with (A) macrophages and (B) bone-marrow derived neutrophils and CFU was recovered as indicated in Materials and Methods in gentamicin protection assays. Values represent Colony Forming Units (CFU) recovered of each strain infecting the phagocytes and expressed as the Ratio of CFU recovered from phagocytes (macrophages and neutrophils) treated vs un treated with cytochalasin D. S. Typhimurium 14028s harvested from cells untreated phagocytes (black bar), S. Typhimurium 14028s harvested from treated phagocytes (white bar), S. Typhimurium ΔarcA harvested from untreated phagocytes (dark blue bar), S. Typhimurium ΔarcA harvested from treated phagocytes (light blue bar), S. Typhimurium ΔarcB from untreated phagocytes (dark grey bar), and S. Typhimurium ΔarcB from treated phagocytes (light grey bar) at 3 hpi. *P < 0.05; **P < 0.01, ***P < 0.001 by one-way ANOVA followed by Bonferroni post hoc test. (TIF) [file pone.0203497.s004.tif]

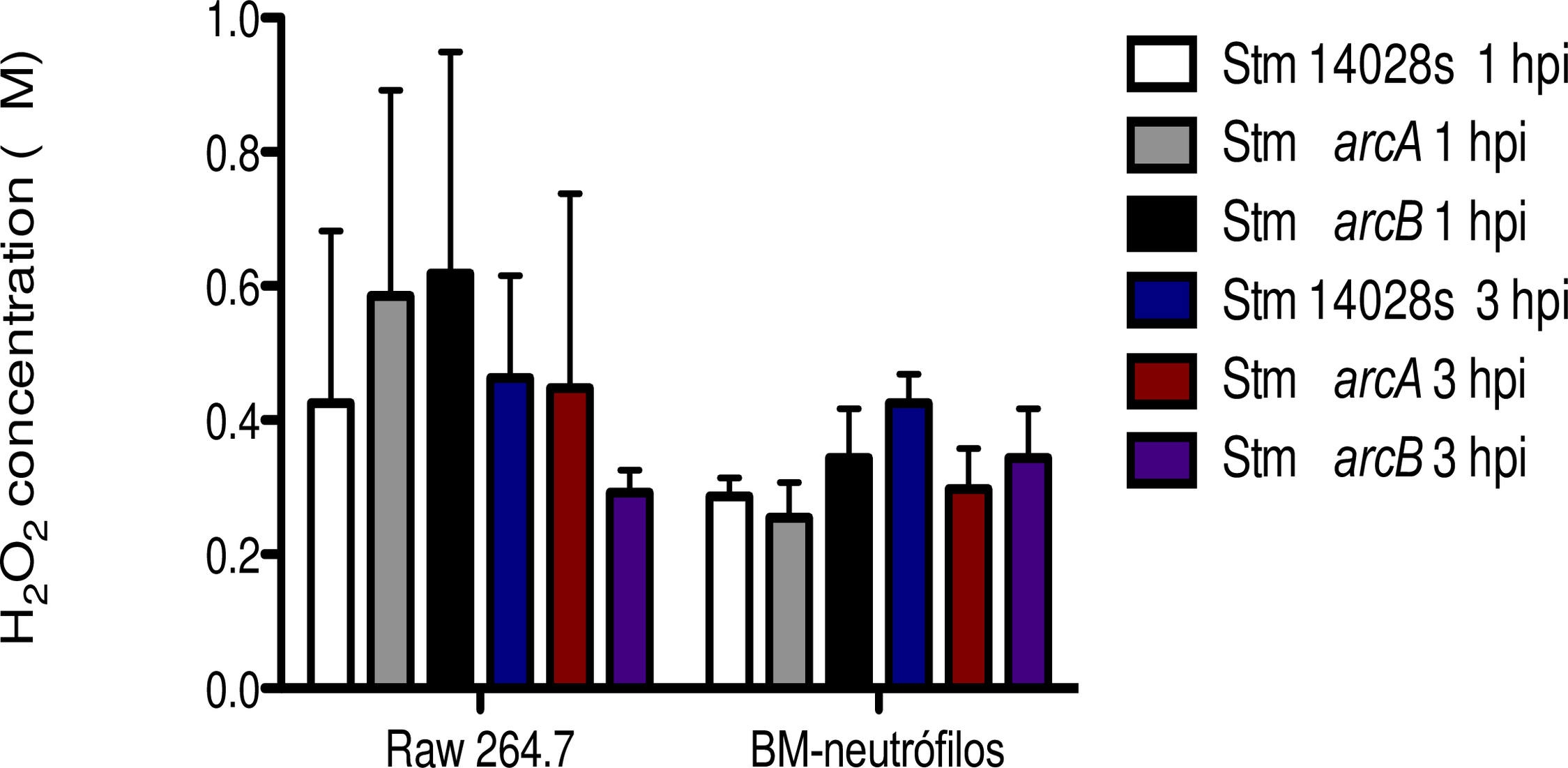

Supplement: S5 Fig — Phagocytic cells were co-cultured with S. Typhimurium 14028s (white bar), S. Typhimurium ΔarcA (grey bar) and S. Typhimurium ΔarcB (black bar) at 1 hpi, and S. Typhimurium 14028s (blue bar), S. Typhimurium ΔarcA (red bar), and S. Typhimurium ΔarcB (purple bar) at 3 hpi. Quantified using Amplex® Red Hydrogen Peroxide/Peroxidase Assay Kit. One-way ANOVA followed by Bonferroni post hoc test., no significate difference found. Data are from 5 biological replicates with 3 technical replicates per experiment. (TIF) [file pone.0203497.s005.tif]

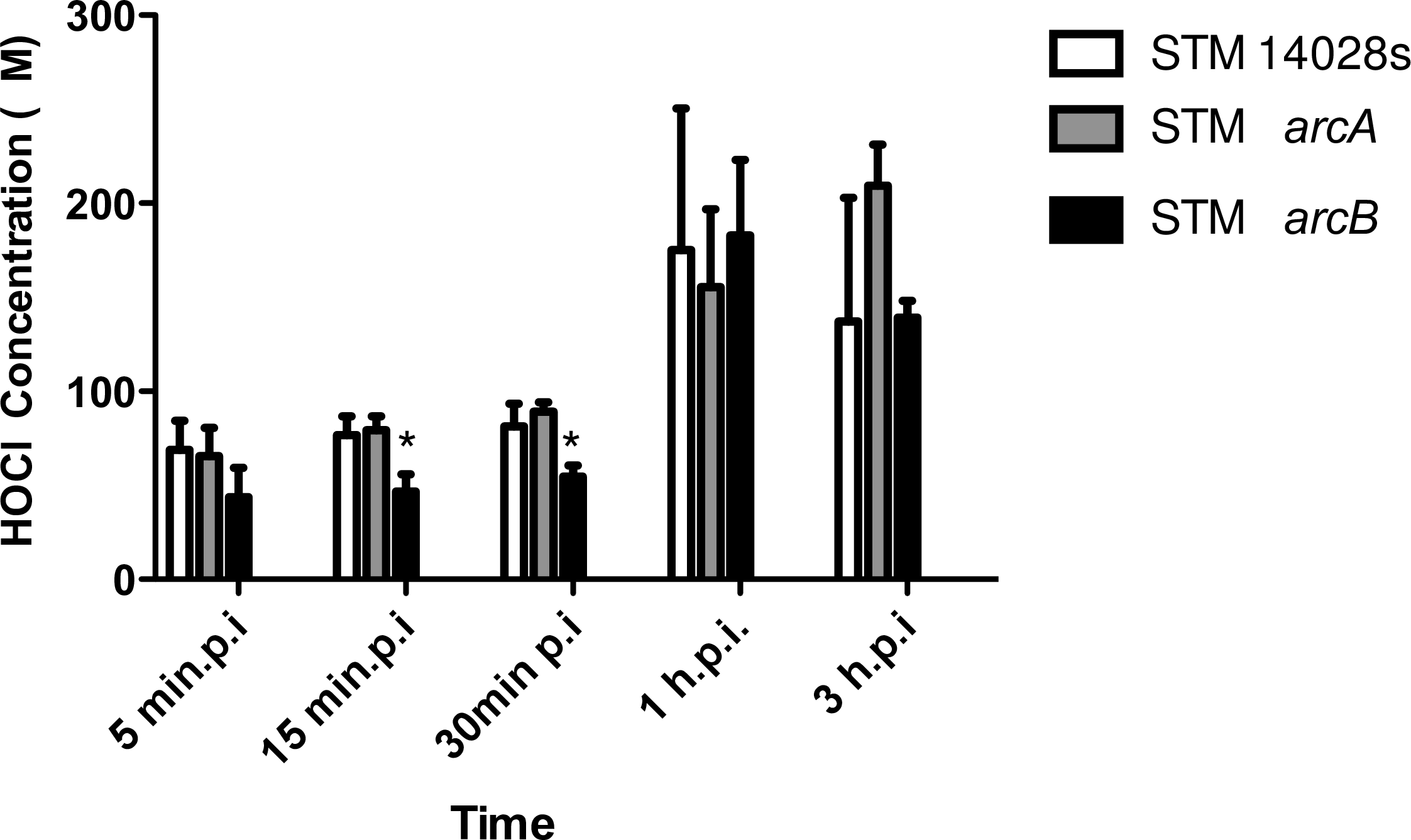

Supplement: S6 Fig — Each phagocyte was co-cultured with S. Typhimurium 14028s/pGLO (white bar), S. Typhimurium ΔarcA/pGLO (grey bar), and S. Typhimurium ΔarcB/pGLO (black bar). The amount of HOCl was quantified by GFP bleaching after cell lysate. *P < 0.05; **P < 0.01, ***P < 0.001 by one-way ANOVA followed by Bonferroni post hoc test. Biological replicates n = 5, 3 technical replicates in each one. Data are from 5 biological replicates with 3 technical replicates per experiment. (TIF) [file pone.0203497.s006.tif]

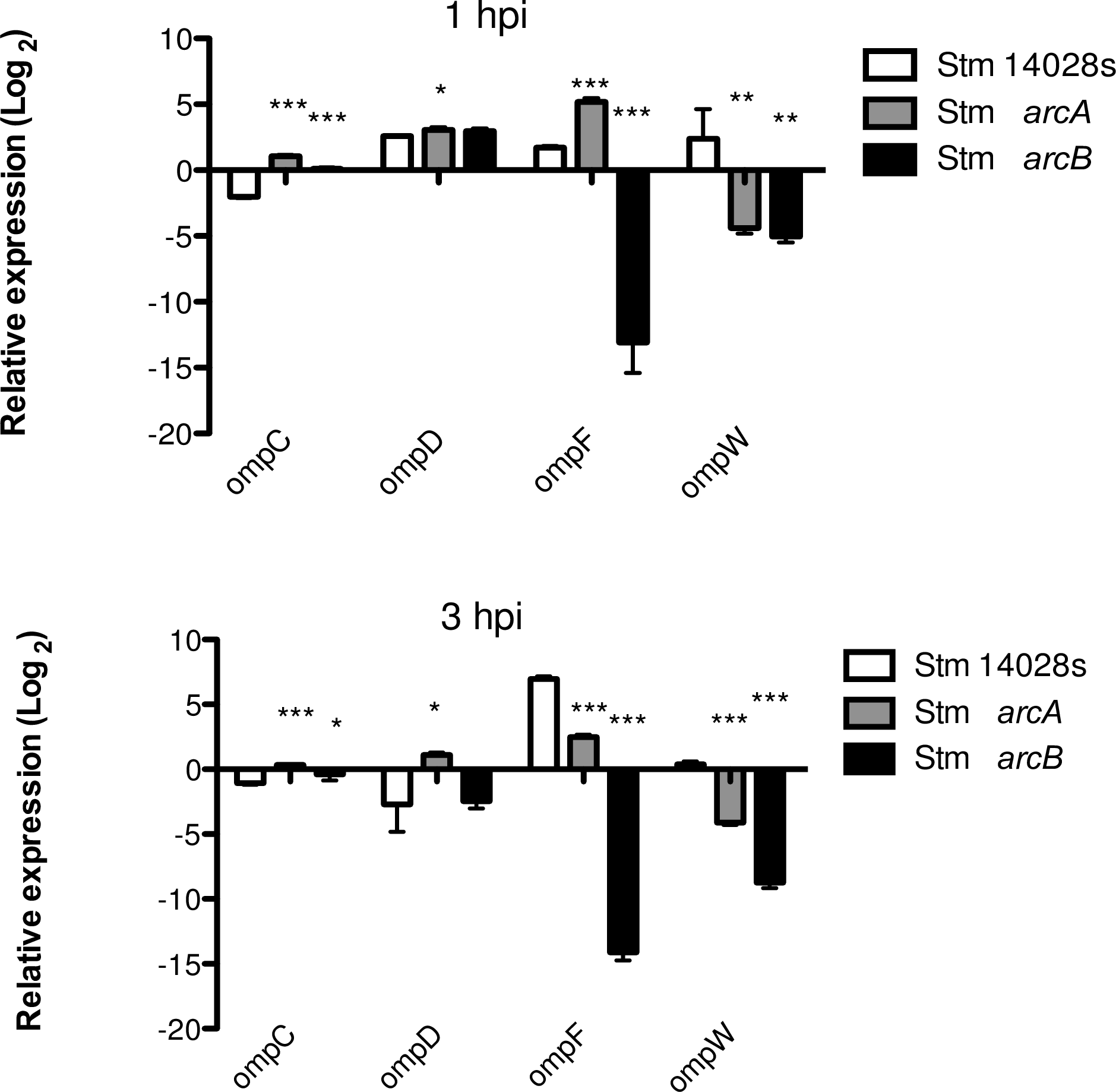

Supplement: S7 Fig — *p<0,05; **p<0,01; ***p<0,001. One Way ANOVA with Bonferroni post-test comparing mutant strains vs wild type strain in 1 hpi and 3 hpi separately. Data are from 5 biological replicates with 3 technical replicates per experiment. (TIF) [file pone.0203497.s007.tif]

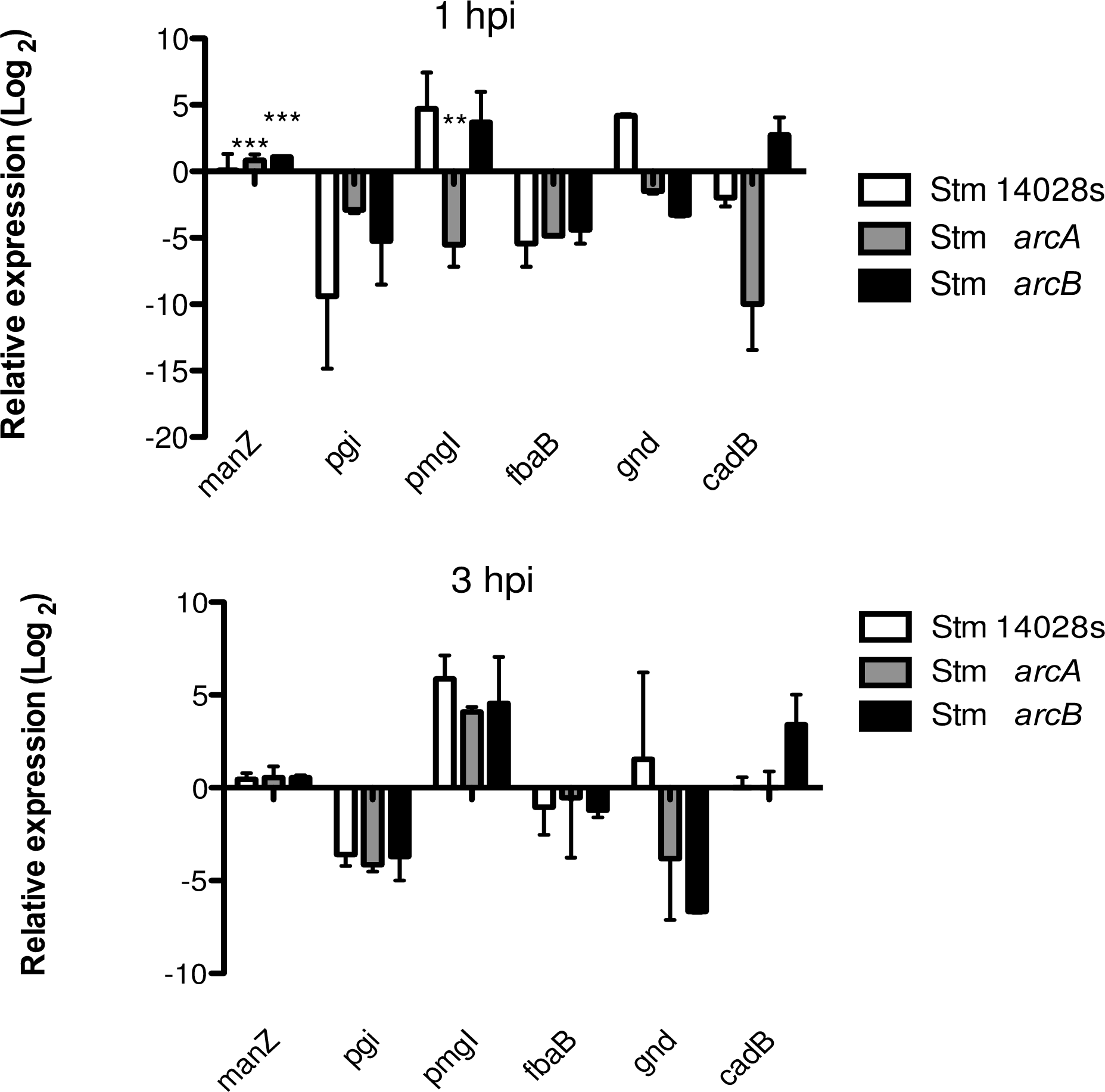

Supplement: S8 Fig — *p<0,05; **p<0,01; ***p<0,001. One Way ANOVA with Bonferroni post-test comparing mutant strains vs wild type strain in 1 hpi and 3 hpi separately. Data are from 5 biological replicates with 3 technical replicates per experiment. (TIF) [file pone.0203497.s008.tif]

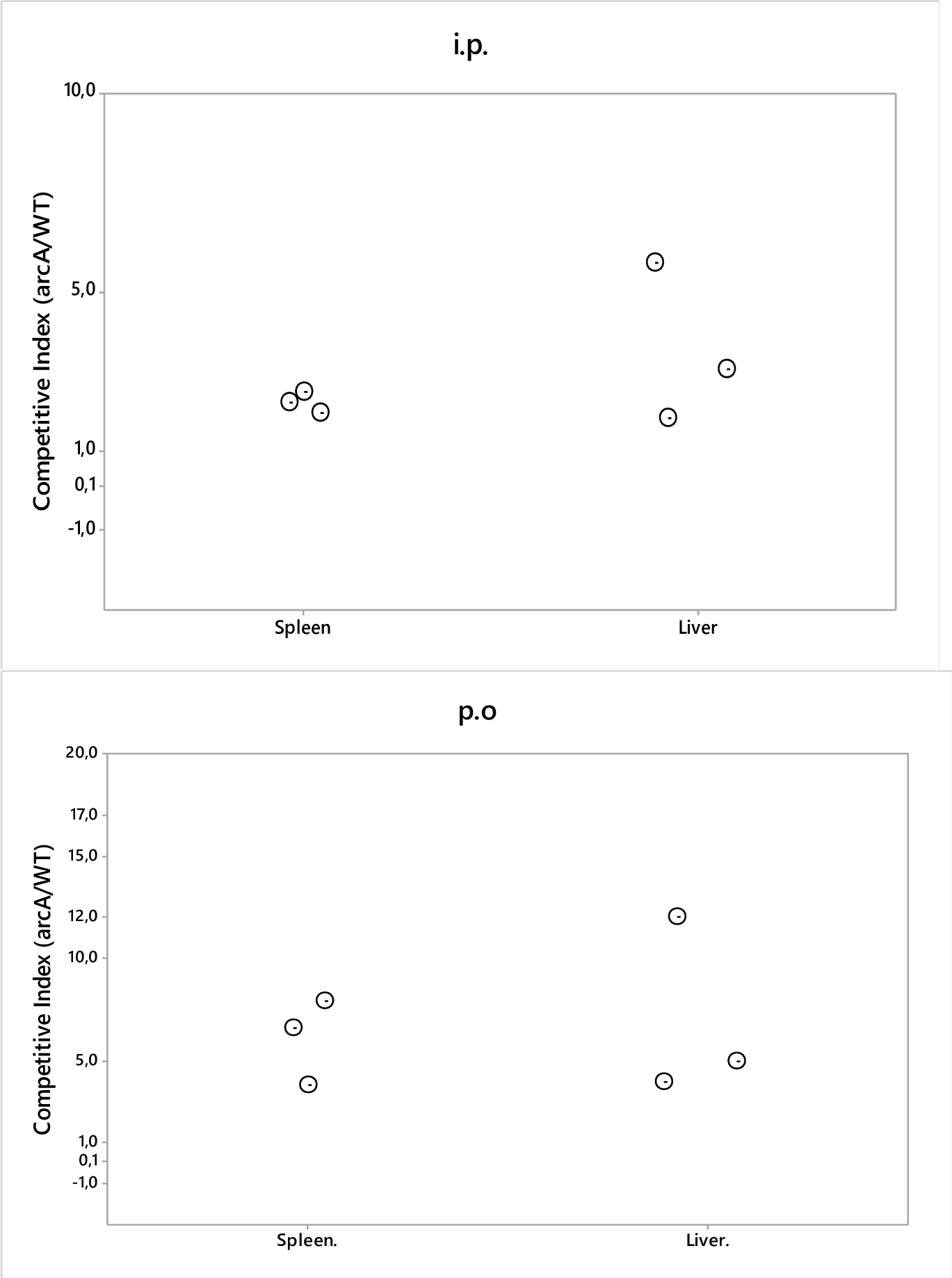

Supplement: S9 Fig — Competitive infections were performed as described before by Evans et al., 2011 [34]. Animals where infected orally (p.o.) of i.p with a 1:1 mixture of S. Typhimurium 14028 and the arcA mutant. Mice were sacrificed at 4 or 6 days p.i and liver and spleen were collected for processing. CI index was calculated as described [34]. (TIF) [file pone.0203497.s009.tif]
