# Supplementary material for: The ArcAB two-component regulatory system promotes resistance to reactive oxygen species and systemic infection by Salmonella Typhimurium
Source: PLoS One. 2018 Sep 4;13(9):e0203497. doi: 10.1371/journal.pone.0203497 (PMC6122832; doi:10.1371/journal.pone.0203497)
Supplement: S1 Table — (PDF) [file pone.0203497.s010.pdf]

Supplementary Table 1. Primers used for qPCR

gene	Primer Forward, sequence (5’-3’)	Primer_Reverse, sequence (5’-3’)
sipC	TGAACGGGCAGAATAGCGTCAA	AGCCTGATGTTCAGGGGAGATTTG
hilA	GTGAAGGGATTATCGCAGTA	CGTAATTGATCCATGAGCTC
sodA	ATGCTAACCACAGCCTGTTCTGGA	ACAGCCAGTTTGTCGCCTTTCA
sodB	AACCACACCTTCTACTGGAACTGC	CCAGTTTGCCATCAGCGCTTTT
sodC	ATGGTCTTACGCCAGGAATTCACG	ATCTGCATTGACAACCAGTCCAGG
katE	ATTCCGGAAGAGTTGGTGCCAGTA	GACGACTGATTTGCGTGTCGGTAT
katG	TTAACTCCTGGCCGGATAAC	TAATCGGCCACAACAAACG
katN	GCGCGAGCGAAGATCATTTAT	GCGACTTCACGGGTCATTAAGA
ahpC	TTTGTTTGCCCGACTGAACTGG	TGTGCGTGAAGTGAGTATCGGT
ahpF	GGCTATCGATCTGGCAGGTATTGT	TACGCACTTTGTCCTGTAGCA
ompC	TACCGTAACACCGACTTCTT	GGTTGCGTTATAGGTCTGAG
ompD	TGCCACCTACCGTAACACT	TTAGACTCGTACGCGCCG
ompF	CACGGCTGGTCATGTAGTTA	AATCTGGTCCGTCTGGCTTT
ompW	ATGAAAAAATTTACAGTGG	GAAACGATAGCCTGCCGA
manZ	GGTATCAAAGTCGGTCTGATGGGA	GGTAGCCATAAGCCACGCCATAAT
pgI	ACGTGGCGAAACACTTTGCT	TTGTCGAAACCGACGGACAGAA
pmgI	ACCGGAAATGAGCTCTGCAGAA	CAACGGACTCAACCGCTTTTGT
fbaB	AATCGTAGCGGCCAGATGGTTG	TTTCGGTTCGGAACAGTCTCGT
talB	ACTATGCGCCAGCTGAAGAT	TCGCTTTCCGCCAGTTCTTT
16s	TACCTGGTCTTGACATCCAC	TTATCACTGGCAGTCTCCTT
arcA	GGCTATGATGTATTCGAAGC	CCGTTGAACTTGTAGCTTTC
arcB	GTGCAAAAGCTGGAGGAGAT	CTCTTGCCGGTTAGCAATTC
